# Application of JAK inhibitors in paradoxical reaction through immune-related dermatoses

**DOI:** 10.3389/fimmu.2024.1341632

**Published:** 2024-02-20

**Authors:** Yaxin Zhang, Guan Jiang

**Affiliations:** ^1^First College for Clinical Medicine, Xuzhou Medical University, Xuzhou, China; ^2^Department of Dermatology, Affiliated Hospital of Xuzhou Medical University, Xuzhou, China

**Keywords:** JAK inhibitors, paradoxical reaction, immune-related dermatoses, atopic dermatitis, psoriasis

## Abstract

Biologics play a positive and effective role in the treatment of immune-related dermatoses. However, many other immune-related diseases have also manifested along with biologics treatment. Paradoxical reaction through immune-related dermatoses refer to the new onset or exacerbation of other immune-mediated dermatoses (mainly psoriasis and atopic dermatitis) after biologics treatment of inflammatory dermatoses (mainly psoriasis and atopic dermatitis), such as new atopic dermatitis (AD) in psoriasis (PsO) treatment and new PsO in AD treatment. A common genetic background and Inflammatory pathway are possible pathogenesis. Faced with paradoxical reactions, the choice of therapy needs to be directed toward therapies effective for both diseases, such as Janus kinase (JAK) inhibitors. The Janus kinase and signal transducer and activator of transcription (JAK-STAT) pathway plays an important role in the inflammatory pathway, and has been widely used in the treatment of AD and PsO in recent years. This article focuses on JAK inhibitors such as tofacitinib, baricitinib, ruxolitinib, Abrocitinib, upadacitinib, and deucravacitinib, to explore the possible application in treatment of paradoxical reactions. Common side effects, baseline risk factors and safety use of JAK inhibitors were discussed.

## 1 Introduction

Paradoxical reaction through immune-related dermatoses refer to the new onset or exacerbation of other immune-mediated dermatoses (mainly psoriasis and atopic dermatitis) after biologics treatment of inflammatory dermatoses (mainly psoriasis and atopic dermatitis) (1). Typical examples are new psoriasis (PsO) in atopic dermatitis (AD) new PsO in AD treatment, inflammation bowel disease (IBD) in PsO treatment, aggravation of systemic lupus erythematosus (SLE) and occurrence of rheumatoid arthritis (RA) (2, 3). The coexistence and transformation between AD and PsO make treatment of these two diseases difficult. In fact, distinguishing between a worsening of the disease because of the lack of drug effect and a paradoxical adverse reaction could be extremely difficult. Moreover, topical corticosteroids can be used for mild to moderate paradoxical reactions, but for severe cases, biologics must be discontinued, which greatly affects the treatment of the primary skin disease. The common genetic background and inflammatory pathways of AD and PsO are possible mechanisms of paradoxical reactions. After the use of biologics, cytokine disorders induce the onset and deterioration of other immune dermatoses (4). Therefore, therapies effective for both diseases are ideal, such as the use of phototherapy, methotrexate, and Janus kinase (JAK) inhibitors. Janus kinase-signal transducers and activators of transcription (JAK-STAT) pathway play a major role in the occurrence and development of immune dermatoses. Targeting intracellular and downstream signaling pathways of cytokines (5–7), it has become a breakthrough in clinical intervention and treatment strategies for both AD and PsO. Currently, JAK inhibitors, such as Abrocitinib, upadatinib and baricitinib, have been widely used. As an effective therapy for both AD and PsO, JAK inhibitors may act on the common inflammatory pathway to treat paradoxical skin reactions.

## 2 Paradoxical dermatoses

### 2.1 Coexistence and transformation of AD and PsO

AD is a common chronic inflammatory dermatosis. There are certain regional and age differences in the incidence of AD with Asian adults ranging from 0.9%-2.1% (8–10) and adults in Europe and the United States 2% to 10% (11). For 1 to 7-year-old children, with a prevalence of 12.94% in China, the prevalence in Europe and the United States is 10% to 30% (11). For adolescents aged 13-14 years, the prevalence in China is 10.1%~15.0% (12), while in Europe and the United States are 4%~21.3% (13, 14). PsO is also a chronic, inflammatory, systemic disease, affecting about 0.1-1.5% of the global population (15). The incidence of PsO varies widely around the world, depending on ethnicity, geographic location, and environment (16).

It has been suggested that AD and PsO cannot coexist in the same person because this requires activation of opposite inflammatory pathways (Th2 vs. Th1) (17). But to date, observational studies support the coexistence and transformation, and paradoxical relationships between other Th2 and Th1 diseases have been reported. Cunliffe et al. conduct observational studies included 20,523 PsO patients and 1,405,911 AD patients. The combined prevalence was 0.3%~12.6%. Prevalence of AD in PSO patients were 17%~20%, and prevalence of PSO in AD patients 0.9%~8.3% (18). Welp et al. found that in 1065 patients with PsO, 8 cases (1.7%) were found to have both PsO and atopic dermatitis (19). Williams found that out of 354 children with AD, 5 (1.4%) had PsO (20).Research by Barry et al. demonstrated that in the group of AD and PsO patients, the proportion of the two diseases coexisting was 1.5%~16.5%. 90% of patients with AD or PsO have an AD or PsO transition, of which, 67% were converted to AD from PsO, 23% from AD to PsO, and 10% co-existing AD and PsO (21).

Dai et al. found that after adjusting for potential confounding factors, patients with AD were at higher risk of PsO (adjusted hazard ratio [aHR] 10.37; 95% CI 6.85–15.69) and the risk of AD in patients with PsO ([aHR] 13.01; 95% CI 10.23–16.56) were higher compared with controls. After excluding patients who had previously used biologics, the association between PsO and AD was similar ([aHR] 13.12; 95% CI 10.31–16.70), indicating that AD and PsO are high-risk factors for each other (22). Simpson et al. found that the standardized prevalence of AD was 1.8 (95% CI, 1.52-2.13) in patients with Th1 disease including PsO, and the prevalence of PsO was 1.36 (95% CI, 1.18-1.56) in patients with Th2 disease including AD, both of which were significantly higher than in the whole population. There was also a significant association between PsO and AD (the standardized prevalence rate for PsO in AD patients was 2.88 [95% CI, 2.38-3.45]) (23).

To conclude above, the coexistence and/or transformation between AD and PsO or vise-versa is with certain percentage of population, but there is no therapy to control paradoxical reaction through immune-related dermatoses, except for biological agents withdrawal and glucocorticoids and cyclosporin application. Withdrawal of biological agents can cause the aggravation of the primary disease, while excessive use of glucocorticoids and cyclosporin has significant side effects. So, new therapy is needed to deal with it. The choice of strategies needs to be directed toward therapies effective for both diseases, such as Janus kinase (JAK) inhibitors.

Moreover, the population within the cohorts may vary with certain parameters including age, region, sex, and many others. Bozek A studied the clinical and immunological characteristics of children with both AD and PsO and compared them to children with only one of these diseases. Patients received oral corticosteroids or immunosuppressants were excluded. Compared with children with a single disease, children with both AD and PsO have unique clinical features: Usually boys and overweight individuals, lesions are usually evenly distributed throughout the body, family history of AD, and serum IL-17 concentrations are significantly higher (24). Patients with these unique clinical features are more likely to have AD and PSO as overlapping syndromes, even without using biological agents. So, for children with unique clinical features having only one disease, JAK inhibitors are suitable, due to their higher possibility to develop paradoxical reaction. Meanwhile, for children already have paradoxical reaction, JAK inhibitors can downregulate their extremely high IL-17 and Th17, at the same time, also downregulate Th22/Th2, avoiding reactive Th22/Th2 increase caused by biological agents.

### 2.2 Pathogenesis of paradoxical dermatoses and the link with biologics

There are specific and co-expressed genes between PsO and AD. Nattkemper LA was found that AD and PsO patients had their specific genes expressed, such as CCL1, IL4, IFN-γ in AD patients and CCL4, IL9, TNFα in PsO patients. But at the same time, AD and PsO patients also have co-expressed genes, such as IL-6/8/17A/22/23A/31 and other cytokines (25). Although most genetic analyses support AD and PsO as opposite diseases, overlapping location or shared cytokines have been noted. There are many common susceptibility sites between PsO and AD, such as chromosomes 1q21, 3q21, 17q25, and 20p. Another shared region is chromosome 5q31.1-q33.1, where IL-13 is associated with both AD and PSO (26). The expression of genes shared by AD and PsO lays the genetic basis for their common inflammatory pathway.

**Figure 1:**
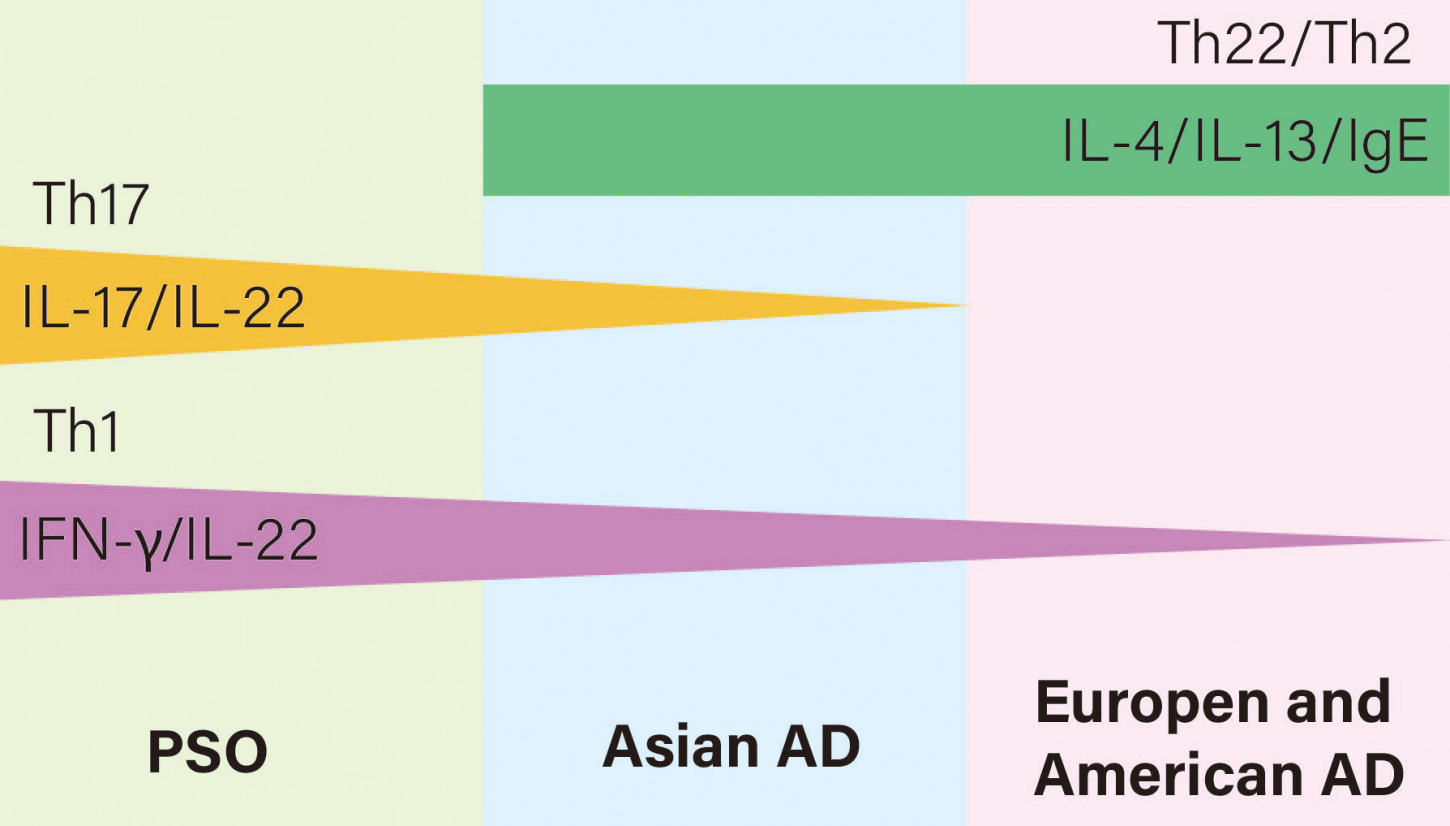
Relationships of T cell polarization and cytokines of AD and PSO on the immune axis. AD is Th22 and Th2 polarized while PsO is Th17 and Th1 polarized. However, AD in Asian populations have both the characteristics of PsO and AD in European and American populations, where Th2, Th17, and Th22 are all activated.

PsO and AD have different core pathogenesis, but they also share a common inflammatory pathway. PsO is a polarized Th1 and Th17 disease with a small amount of Th22 activation, associated with cytokines such as IFN-γ, IL-17, IL-22, etc. AD, on the other hand, is polarized by Th2 and Th22, with a small amount of Th1 activation in the chronic phase, mainly with IL-4, IL-13 and IgE, as detailed in **Table 1**. Moy AP et al. found that the ratio of Th1:Th2 in patients with chronic PsO was significantly higher than that in patients with chronic AD (27). However, we can suggest the assumption that AD and PsO exist in the same lineage, in which they have different T cell polarities, but also produce some overlapping features. For example, AD in Asian populations have both the characteristics of PsO and AD in European and American populations, where Th2, Th17, and Th22 are all activated at the lesions (28), as shown in **Figure 1**. The application of biologics can lead to changes in T cell polarity and induce paradoxical dermatoses, such as the control of Th2 cells may lead to the transformation into Th1 and Th17 cells, thus causing PsO (29). Inhibiting Th1/Th17 cells may lead to conversion to Th2 cells, causing AD.

**Table 1:** Different core pathogenesis of AD and PsO as well as JAK inhibitors.

Location	AD	PsO
In skin lesions	Polarization: Th2/Th22 Th1(a small amount in the chronic phase)	Polarization: Th1/Th17 Th22(a small amount)
	Key and core factors: IL-4 and IL-13	Key and core factors: IL-23 and IL-17
	Th17 pathway downregulated	Th17 pathway upregulated
	Antimicrobial factor synthesis is decreased	Antimicrobial factor synthesis is increased
In blood circulation	TH2 > TH1 and TH17	TH1, TH17 and TH22 increase
	IgE levels and eosinophil counts increase	lgE levels and eosinophil counts are normal
	lgE autoantibodies are associated with disease activity	Autoantibodies are uncommon
JAK Inhibitors (oral)	tofacitinib (JAK1/3) delgatinib (pan-JAK) deucravacitinib (JAK1/TYK2) baricitinib (JAK1/2) upadacitinib (JAK1) abrocitinib (JAK1)	tofacitinib (JAK1/3) peficitinib (pan-JAK) baricitinib (JAK1/2) solcitinib (JAK1) itacitinib (JAK1)
JAK Inhibitors (topical)	tofacitinib (JAK1/3) ruxolitinib (JAK1/2)	tofacitinib (JAK1/3)

Biologics play a positive and effective role in the treatment of PsO and AD. However, paradoxical reactions to many immune-related dermatological diseases arise with the application of biologics. A systematic review of 2049 paradoxical skin reactions found that: The paradoxical skin reactions caused by TNF-α inhibitors and IL-17 inhibitors accounted for 91.2% and 3.5% respectively, while IL12/23 inhibitors accounted for 2.4% and IL-23 inhibitors accounted for 0.01% (26). TNF-α inhibitors, such as adalimumab and infliximab, are used to treat PsO and hidradenitis suppurativa (30). It leads to paradoxical PsO, possibly due to the fact that TNF-α signaling inhibition can alleviate the TNF-α -dependent negative feedback of plasmacytoid dendritic cells, resulting in an overproduction of type I interferon, activating the Th1 and Th17 axes causing PsO (31). IL-17 inhibitors, such as secukijo and ixekizumab, used to treat PsO, can induce AD-like rash. The possible cause is that the inhibition of Th1/Th17 phenotype of PsO shift the immune balance to the Th2 phenotype of AD (4). Meanwhile, IL-17 inhibitors can inhibit the repair effect of IL-17 on skin barrier function, resulting in a decrease in the production of antimicrobial peptides by keratinocytes, increasing colonization of Staphylococcus aureus or fungi, and then inducing AD-like damage. IL-23 inhibitors, such as ustekinumab, can be used in the treatment of PsO. By reducing IL-17 secretion and Th17 differentiation, it inhibits the skin inflammatory response. But this inhibition can also lead to paradoxical reactions, which in turn induces the production of AD-like rash (32). IL-4R inhibitors, such as dupilumab, are used in the treatment of AD. Dupilumab can also cause paradoxical reactions, such as PsO-like lesions, but occurs less frequently than other biologics. In clinical trials and long-term extended safety studies, only 0.3% of adults with moderate-to-severe AD report adverse effects of PsO-like rash during treatment (33). This may be due to the fact that dupilumab selectively inhibits Th2 cytokines without affecting Th1/17/22 cytokine levels (34). Moreover, infections, a common side-effect of immunosuppression treatment, might paradoxically act as an immunostimulatory trigger for autoimmune disorders (35).

## 3 JAK inhibitors

### 3.1 JAK-STAT signaling pathway and JAK inhibitors

Janus kinase-the signal transducer and activator of transcription (JAK-STAT) signaling pathway plays a major role in the intracellular signaling of most discovered cytokines in recent years. Dysregulation of the JAK-STAT pathway plays a key role in the pathophysiology of some cytokine-dependent autoimmune and inflammatory dermatoses, such as AD and systemic lupus erythematosus (SLE) (36, 37). Targeting cytokines and downstream signaling pathways has become an important research area. JAK inhibitors in the treatment of inflammatory-mediated dermatoses have rapidly become popular and have made significant contributions to clinical medicine (38).

Janus kinases are intracellular non-receptor tyrosine kinases. JAKs can bind to and phosphorylate cytokine receptors and phosphorylate multiple signaling molecules containing SH2 domains. Each JAK structure has seven homology regions (JH1-7) that make up four distinct domains: FERM, SH2, and classical protein tyrosine kinase (PTK) and pseudo kinases (39). Among them, FERM and SH2 structure domains are involved in binding JAK and its cognate receptors (40, 41). The classical protein tyrosine kinase (PTK) domain is a catalytic domain that is primarily responsible for phosphorylation of cytokine receptors and STAT proteins (42). The function of the pseudokinase domain remains largely unknown, but it is increasingly thought to play an important regulatory role in the function of phosphorylation (43).

Cytokines induce receptor dimerization by binding to their cell surface homologous receptors and then activating JAKs. Activated JAKs phosphorylate tyrosine residues on the receptor catalytic domain and form docking sites, thereby recruiting STAT proteins carrying the SH2 domain. Subsequently, the STAT protein recruited to the docking site is phosphorylated and induced to form a dimer that can translocate the nucleus and bind to specific elements on DNA to regulate downstream gene transcription (44). Therefore, the JAK-STAT pathway, which consists of JAKs and STAT, can directly link cell surface events with gene transcriptional regulation in the nucleus and is regarded as the central communication point for cellular function. In principle, many drugs can target the JAK signaling pathway, but this review has focused on JAK inhibitors.

JAK inhibitors block the JAK-STAT signaling pathway by inhibiting JAK activity and blocking signaling, phosphorylation, and activation of transcriptional activators. In humans, there are four members of the JAK family: JAK1, JAK2, JAK3, and tyrosine kinase 2 (TYK2). Most cytokines act through a combination of JAK members, so the blocking effect of JAK inhibitors is complex.

JAK1 plays a key role in the classical signaling pathway of type I and type II interferons (45). JAK1 inhibitors can significantly block key inflammatory signaling pathways downstream of IL-6 and IFNα/β/γ, and protect joints by improving inflammation (46). JAK2 is closely related to receptor signaling pathways, such as erythropoietin, thrombopoietin, and hematopoietic growth factors. JAK2 inhibitors can block granulocyte-macrophage colony-stimulating factor (GM-CSF)-induced inflammation, resulting in dysfunction of hematopoietic cell proliferation, differentiation, and functional activation (47). These blocking effects exacerbate aggravation stunting and anemia caused by chronic diseases (46) (48, 49). In contrast to the widespread distribution of JAK1, JAK2, and TYK2, JAK3 is predominantly expressed in hematopoietic cells. In addition, the γ chain (γc) subunits of some cytokine receptors have been specifically selected to conduct intracellular signaling coupled to JAK3, including IL-2, IL-4, IL-7, IL-9, and IL-15. In a JAK3- knockout mouse model, mice exhibited lymphocytosis and impaired immune function (50). JAK3 inhibitors has been shown to suppress immune cells (such as NK cells), downregulate immune function, and reduce the body’s defenses against infections and tumors (46, 49, 51). One study showed that TYK2-deficient mice exhibited down-regulated signaling responses to IFN-α and IL-12, suggesting that anti-TYK2 has the potential to affect the maturation of innate immune cells and adaptive immune cells, increasing the risk of viral infection and recurrence (52).

### 3.2 The role of the JAK-STAT pathway in paradoxical reactions and the possible mechanism of JAK inhibitors in the treatment

Th2-associated cytokines, including IL-4, IL-5, IL-13, and IL-31, play a key role in the development of AD lesions (53). These cytokines are produced by Th2 and act on Th2 itself, and each factor promotes each other to produce a large number of cytokines through positive feedback. IL-22 and IL-20 can proliferate spinous layer of epidermis. The combined effect of IL-22, IL-4, and IL-13 promotes keratosis, which ultimately leads to damage of the skin barrier (54).

The Th2 immune response is associated with the upregulation of all four JAKs. Th2-related cytokines rely on the JAK pathway to stimulate the production of more cytokines. There are two kinds of IL-4 receptor. Binding to type 1 receptors results in phosphorylation of JAK1 and JAK3, leading to STAT6 activation, while binding to type 2 receptors induces JAK1 and TYK2 expression, activating STAT6 and STAT3 (53). The IL-5-mediated pathway transduce its signaling through JAK1 and JAK2 as well as STAT1, STAT2, and STAT5 (55–57). IL-31 and intracellular signaling of thymic stromal lymphopoietin (TSLP) are transmitted by JAK1 and JAK2, followed by STAT1, STAT3, and STAT5 (58, 59), which also promotes Th2 differentiation.

JAK/STAT-dependent IL-4 and IL-13 signaling is also important in dysfunction of AD keratinocytes (58). For example, IL-4 and IL-13 downregulate loricrin and involucrin in keratinocytes, which are structural proteins that contribute to the protective barrier function of the stratum corneum. In addition, downstream signaling of IL-4 and IL-13 modulates the expression of innate immune response genes, including cathelicidin and β-defensin, increasing sensitivity to skin infections (such as Staphylococcus aureus), which in turn act on keratinocytes and immune cells to exacerbate AD (60–62).

While the main cytokine involved in the pathogenesis of AD is the Th2 cytokine, the Th1 and Th17 immune responses may also play a role in certain AD subtypes and chronic AD lesions (63). The hallmark cytokine of Th1 is IFN-γ, a protein that utilizes STAT1 for signal transduction. In addition, STAT4 is required for IL-12 signaling, which differentiates naïve T helper cells into Th53 cells (64).

At the heart of the pathogenesis of PsO are cytokines on the IL-17/IL-23 axis. The JAK-STAT pathway regulates the intracellular signaling of several cytokines on the IL-12/IL-23 axis, including IL-6, 17, 22, 23, and IFN-γ. The hallmark cytokine of Th1 is IFN-γ, a protein that utilizes STAT1 for signal transduction. STAT4 is also required for IL-12 signaling. IL-23 mediate its action through a cell receptor complex, which is composed of two transmembrane proteins: IL-12Rβ1 and IL-23Rα, Extracellular domain binds to IL-23, while inner domain binds to TYK2 and JAK2. These tyrosine kinases transfer phosphate groups to tyrosine residues of intracellular signaling molecules, thereby propagating cytokine binding signals from extracellular to intracellular (65).

### 3.3 Clinical trials of JAK inhibitors in AD and PsO treatment

The JAK-STAT pathway plays a key role in the pathogenesis of AD and PsO. Therefore, JAK inhibitors have the potential to treat paradoxical dermatoses. At present, clinical trials have shown that the JAK inhibitor that can treat AD is tofacitinib (JAK1/3), ruxolitinib (JAK1/2), delgatinib (pan-JAK), deucravacitinib (JAK1/TYK2), baricitinib (JAK1/2), upadacitinib (JAK1), Abrocitinib (JAK1), by blocking cytokines of the Th2 axis such as IL-4, IL-5, IL-13, IL-31 (66). Pso can be treated by Tofacitinib (JAK1/3),peficitinib, (pan-JAK); baricitinib(JAK1/2)solcitinib (JAK1); itacitinib (JAK1) blocking cytokines on Th1, Th17, and Th22 axes such as IL-17/IL-23 (67). Clinical trials have shown in **Table 2**.

**Table 2:** Clinical trials of JAK inhibitos of treating AD and PSO.

JAK Inhibitor	Diseases	Research phase	Number of patients	Duration (weeks)	Dose	Primary Endpoints Met
Tofacitinib (JAK1/3)(topical)	AD	Phase 2a	69	4	2% BID	%EASI
Tofacitinib (JAK1/3)(oral)	PSO	Phase 2b	197	12	2mg, 5mg, 15mg, BID	PASI75
		Phase 3	901 + 960	16	5mg,10mg, BID	PASI75, PGA (0 or 1)
		Phase 3	1106	12	5mg,10mg, BID	PASI75, PGA (0 or 1)
		Phase 3	666	24	5mg,10mg, BID	PASI75, PGA (0 or 1)
		Phase 3	266	36	5mg,10mg, BID	PASI75, PGA (0 or 1)
Ruxolitinib (JAK1/2)(topical)	AD	Phase 2b	307	12	1.5% BID, 1.5% QD, 0.5% QD, 0.15% QD	%EASI
		Phase 3	631	52	1.5% BID, 0.75% BID	IGA 0/1
		Phase 3	618	52	1.5% BID, 0.75% BID	IGA 0/1
Delgocitinib (JAK1/2/3/TYK2)(topical)	AD	Phase 2	327	4	3% BID, 1% BID, 0.5% BID, 0.25% BID	%EASI
		Phase 2	103	4	0.5% BID, 0.25% BID	%EASI
		Phase 3	158	28	0.5% BID	%EASI
		Phase 3	352	52	0.5% BID	Safety
Baricitinib (JAK1/2)(oral)	AD	Phase 2b	124	16	4mg, 2mg daily dose	EASI-50 (4 mg only)
		Phase 3	624	16	4mg, 2mg,1mg daily dose	vIGA-AD 0/1 (only 4 and 2 mg)
		Phase 3	615	16	4mg, 2mg,1mg daily dose	vIGA-AD 0/1 (only 4 and 2 mg)
		Phase 3	Enrolling	52	4mg, 2mg,1mg daily dose	vIGA-AD 0/1
		Phase 3	463	16	4mg, 2mg,1mg daily dose	EASI-75 (4 mg)
		Phase 3	440	16	2mg,1mg daily dose	EASI-75
		Phase 3	Enrolling	16 + variable	4mg, 2mg daily dose	EASI-75
		Phase 3	329	16	4mg, 2mg daily dose	vIGA-AD 0/1 (only 4 mg)
Baricitinib (JAK1/2)(oral)	PSO	Phase 2b	238	12	10mg, 8mg, 4mg, 2mg daily dose	PASI75
Abrocitinib(JAK1)(oral)	AD	Phase 2b	267	12	200mg, 100mg, 30mg, 10mg daily	IGA 0/1 (only 200 and 100 mg)
		Phase 3	387	12	200mg, 100mg daily	IGA 0/1,EASI-75
		Phase 3	391	12	200mg, 100mg daily	IGA 0/1,EASI-75
		Phase 3	838	16	200mg, 100mg daily	IGA 0/1,EASI-75
		Phase 3	285	12	200mg, 100mg daily	IGA 0/1,EASI-75
		Phase 3	1,233	52	200mg, 100mg daily	EASI-50, IGA
		Phase 3	Enrolling	92 + variable	200mg, 100mg daily	Safety
solcitinib (JAK1) (oral)	PSO	Phase 2a	60	12	400mg, 200mg, 100mg daily	PASI75
itacitinib (JAK1)(oral)	PSO	Phase 2	50	4	600mg,400mg, 200mg, 100mg daily	Mean PGA reduction from baseline

In multiple clinical trials, tofacitinib (JAK1/3), baricitinib(JAK1/2)and ruxolitinib(JAK1/2)have been tested for the treatment of both AD and PsO, with the effect of preventing and treating paradoxical skin reactions. Topical tofacitinib have been evaluated for the treatment of mild to moderate AD. In a 4-week phase 2 randomized double-blind controlled trial (RDBVCT) in adults aged 18-60 years, the mean proportion of Percentage change from baseline (CFB) in Eczema Area and Severity Index (EASI) score was significantly greater for tofacitinib (-81.7%) vs. vehicle (-29.9%) (68). In addition, oral tofacitinib have shown reasonable efficacy for moderate to severe AD. In a number of case reports and case series, a total of 9 patients with moderate to severe AD had significant efficacy. Decreased body surface area involvement of dermatitis and decreased erythema, edema/papulation, lichenification, and excoriation were observed in all patients, with no adverse effects (69–72). At the same time, tofacitinib is the most studied JAK inhibitors for chronic plaque PsO. We found five clinical trials of tofacitinib for PsO, including one Phase 2 trial and four Phase 3 trials (73–77). Significant improvements in Psoriasis area and severity index (PASI)-75, Dermatology Life Quality Index (DLQI), Nail PsO Severity Index (NAPSI), and PASI-90 response were also observed (73).

Baricitinib, oral JAK1/2 inhibitors, originally approved by the FDA in 2018 for the treatment of moderate to severe rheumatoid arthritis in adults. We found 8 clinical trials for baricitinib treating AD, including one phase 2 trial and seven phase 3 trials, all of which had significant efficacy and ideal safety (78–83). In 2020, it was approved in Europe and Japan for the treatment of moderate to severe AD. It has also shown significant efficacy in PsO. Baricitinib has been tested as treatment for PsO in a phase 2 clinical trial. In a 12-week phase 2 randomized double-blind controlled trial (RDBVCT) of 238 North American patients, the proportion of PASI-75 response was significantly greater for baricitinib (8mg) (43%) vs. baricitinib (8mg) (54%) vs. control group (17%).

Similar to baricitinib, ruxolitinib is JAK1/2 inhibitors, which was first approved by the FDA in 2011 for the treatment of myelofibrosis, and later added erythrocytosis vera and acute graft-versus-host reaction. Despite oral ruxolitinib has not been tested to treat AD, topical formulations are expected for mild to moderate AD. In a randomized, double-blind, controlled, phase II clinical trial lasting 8 weeks (84, 85), the proportion of EASI was significantly greater for RUX 1.5% ointment BID (71.6%) vs. control group (8mg) (15.5%). In another randomized, double-blind, controlled, phase III clinical trial lasting 8 weeks (86), ≥ 12-year-old patients with mild to moderate AD were enrolled. The proportion of IGA 0/1 was significantly greater for RUX 1.5% cream (53.8%) vs. RUX 0.75% cream (50.0%) vs. control group (15.1%).

Similarly, for PsO, topical ruxolitinib has also been tested in a number of phase 2 clinical trials (87). After application of ruxolitinib, the mean lesion score and area continued to decrease, with a greater reduction compared to the control lesions. Different doses (1.5%BID, 1.5%QD,1.0%BID)had similar efficacy, with an average reduction of 42%-55% in erythema, 46%-78% in scale, 50%−65% in thickness.For the above three JAK inhibitors, tofacitinib (JAK1/3), baricitinib(JAK1/2)and ruxolitinib(JAK1/2)all have been tested in clinical trials for the treatment of AD and PsO, and have the potential to treat paradoxical reactions.

Abrocitinib, upadacitinib, solcitinib, and itacitinib are all JAK1 inhibitors, among which abrocitinib and upadacitinib have been tested in clinical trials for AD, while solcitinib and itacitinib are used for the treatment of PsO. We found seven clinical trials for abrocitinib applied in AD, including a phase 2 trial and six phase 3 trials, all having significant efficacy and good safety, with primary endpoints achievement rate of IGA0/1 and EASI-75 response significantly higher than those in the control group (88–91). Similarly, upadacitinib have been used to treat AD. There were five clinical trials, one phase 2 trial and four phase 3 trials. The arrival rate of the primary endpoints of EASI75 and IGA0/1 was significantly higher than that of the control group (92). Although there are no clinical trials for the treatment of PsO, upadacitinib has been approved for the treatment of psoriatic arthritis by the mechanism to inhibit cytokine such as IL-6 and IL-23. For the treatment of AD, abrocitinib and upadacitinib have been tested by extensive clinical trials for their efficacy and safety.

Solcitinib and itacitinib are two new types of JAK1 inhibitors, with few clinical trials at present. Currently there is only one phase 2 clinical trial for the treatment of PsO. In the 12-week clinical trials of solcitinib (GSK2586184) (93), the PASI75 response rate at week 12 was significantly different in the 400 mg group than in the placebo group. And a dose-response relationship was observed:13%(100mg), 25%(200mg), 57%(400mg), 0%(placebo). In a clinical trial of itacitinib (INCB039110) for 28 days (94), compared to placebo, the mean percentage reduction in Patients’ Global Assessment (PGA) was significantly improved in the 200 mg twice daily and 600 mg daily groups. For itacitinib, there were no serious adverse events, but for solcitinib, five serious adverse events were reported, two of which were thought to be related to solcitinib treatment, included one case of ureteral stones and one case of severe thrombocytopenia, with safety still needed to be considered.

**Figure 2:**
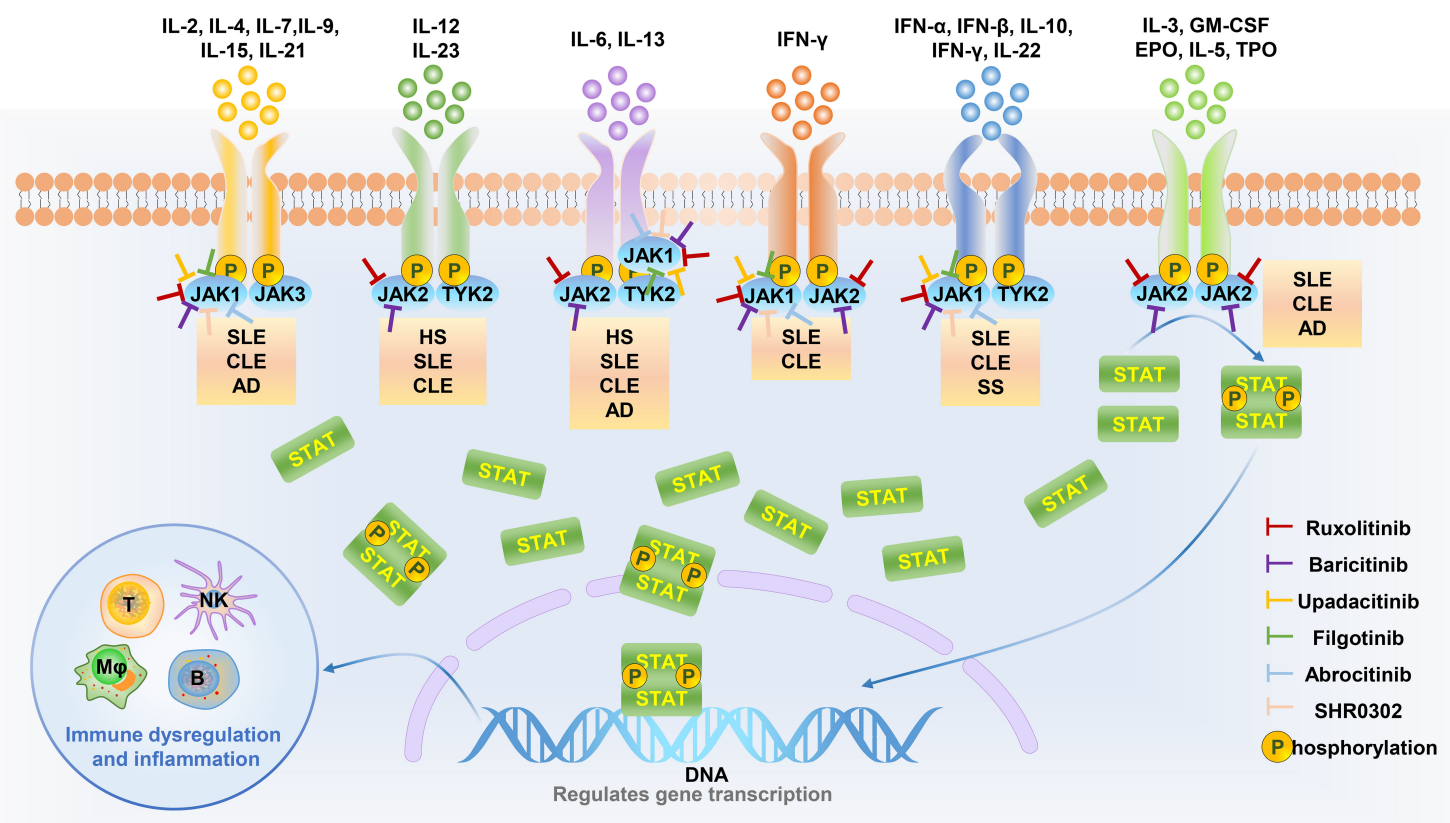
JAK inhibitors target specific JAK kinases to inhibit skin disease-related cytokine downstream signaling pathways. Different cytokines are involved in the pathological processes of different diseases through signals mediated by specific JAK kinases. JAK inhibitors can be used as a therapeutic strategy for cytokine-related diseases by inhibiting specific JAK kinase activity. HS, Hidradenitis suppurativa; SLE, Systemic lupus erythematosus; CLE, Cutaneous lupus erythematosus; AD, Atopic dermatitis; SS, Sjögren’s syndrome.

Abrocitinib and upadacitinib have been tested in clinical trials for the treatment of AD, while sucitinib and itatinib have been used for the treatment of PsO. Abrocitinib, upadatinib, solcitinib, and itacitinib are all JAK1 inhibitors, the target spot is consistent. It is speculated that upadatinib and abrocitinib can work in the treatment of PSO and solcitinib and itacitinib can work in the treatment of AD. The hypothetical mechanism are as follows: In the development of AD lesions, Th2-related cytokines, including IL-4, IL-5, IL-13, and IL-31, play a key role (53). IL-4 receptors are coupled to JAK1 and JAK3, and IL-13 receptors are coupled to JAK1/2 and TYK2. Blocking JAK1 can inhibit the occurrence and progression of AD. The core pathogenesis of PsO is cytokines on the IL-17/IL-23 axis, including IL-6, 17, 22, 23 and IFN-γ. IL-6 receptors are coupled to JAK1/2 and TYK2, while IL-22 and IFN-γ receptor are coupled to JAK1/TYK2. Blocking JAK1 can also inhibit the occurrence and development of PSO. Therefore, JAK1 inhibitors have a hypothetical mechanism for the simultaneous treatment of AD and PSO, as shown in **Figure 2**. Therefore, Abrocitinib, upadatinib, solcitinib, and itacitinib have the potential to treat paradoxical dermatoses, but clinical trials need to be conducted to test their efficacy and safety, especially for the less studied solcitinib and itacitinib (95).

Deucravacitinib (THICK2) was approved by the FDA in 2022 for the treatment of moderate to severe plaque PsO (96). There was a phase 2 and two phase 3 clinical trials on deucravacitinib for the treatment of PsO (97, 98). In one of the phase II multicenter, double-blind trials. the incidence of PASI75 was significantly higher in patients received deucravacitinib at a daily dose of 3 mg or higher than in the placebo group. In one of the randomized, double-blind controlled phase 3 clinical trials, the response rate to the primary endpoint PASI75 at 16 weeks, 6 mg QD was significantly higher than that of the control group (58.4%vs12.7), PGA 0/1 was also significantly higher than in the control group (53.6%vs7.2%).

As a TYK2 inhibitor, deucravacitinib is used for the treatment of PsO. However, at the same time, TYK2 also plays an important role in the pathogenesis of AD, so it is speculated that it has a therapeutic role in AD. The hypothetical mechanism is as follows. Th2-related cytokines, including IL-4, IL-5, IL-13, and IL-31, play an important role in the pathogenesis of AD (53). IL-13 can downregulate structural proteins such as loricrin and involucrin in keratinocytes which contribute to the protective barrier function of the stratum corneum, thus promote dyskeratosis. At the same time, modulating genes for innate immune responses including cathelicidin and β-defensin, IL-13 enhance susceptibility to skin infections such as Staphylococcus aureus to destroy the skin barrier, promoting the development of AD (60–62). IL-13 receptor is coupled to TYK2 and JAK1/2, so TYK2 inhibitors can block the effect of IL-13 to develop AD, as shown in **Figure 2**. However, animal experiments and clinical trials are still needed to confirm its efficacy and safety.

### 3.4 The use of JAK inhibitors in other paradoxical reaction

Except for AD and PsO, many cases of other immune-related diseases also manifest along, such as inflammatory bowel disease in psoriasis, exacerbation of SLE and occurrence of rheumatoid arthritis.

Inflammatory bowel disease (IBD) is a common immune-related diseases treated by TNFα and IL-17 inhibitors (99). However, it can also be paradoxically induced by these biologics in psoriasis treatment. Since it is IL-17F contributes to colitis, while IL-17A acts at the mucosal interface, maintaining and protecting the epithelial barrier (100). JAK inhibitors are new approaches for IBD, with Tofacitinib already approved and others in Phase II/III recruiting (101). Notably, excessive production of pro inflammatory cytokines, including IL-6, IL-23, IL-12 and IL-21 contributes to the incidence of IBD, while JAK inhibitors could block the pathway and downregulate the cytokines (102). As a result, JAK inhibitors are promised drugs to treat paradoxical reaction of IBD, as well as primary disease of psoriasis. Moreover, though there are currently no data available on the combination of a JAK inhibitor and biologics in IBD, it could be a potentially appealing and safe approach.

Systemic lupus erythematosus (SLE) is a condition in which the immune system activation is characterized by exaggerated B cell and T cell responses and loss of immune tolerance against self-antigens. Biologics aiming to target specific molecular is promised treatment of SLE, but cases of paradoxical reactions have been reported. Lupus-like paradoxical reaction from TNFai vary from systemic lupus erythematosus (SLE) to lupus-like syndromes to isolated cutaneous lupus, with the most commonly reported inciting drugs of infliximab (56%), adalimumab (25%), and etanercept (15.5%) with incidence rates of 0.175%, 0.06%, and 0.09%, respectively (103). In literature review, 25 cases with rheumatic diseases of biologics and targeted synthetic drugs inducing immune−mediated glomerular disorders were found (104, 105). A mechanism linked to their proteinic structure has been hypothesized. Anti-TNFα agents might bind to immune cell products, determining the formation of immunocomplexes or inducing inflammatory cell apoptosis, which causes the release of immunogenic nucleosome antigens (105). TNFα inhibition also exerts a direct effect on lymphocyte function and cytokine production, switching from Th1 to Th2 (106). Moreover, infections, a common side-effect of immunosuppression treatment, might paradoxically act as an immunostimulatory trigger for autoimmune disorders (35). Since many biologicals such as CD20 antibodies targeting B cells have appeared promising but not yield favorable, low‐molecular‐weight compounds is expected. Baricitinib was used in a phase IIb clinical trial for patients with SLE, which is promising (107). JAK inhibitors could block the swift from Th1 to Th2, possibly reducing the incidence of paradoxical reaction.

New treatment strategies such as biologics have substantially changed the course of rheumatoid arthritis (RA). Biologics such as Adalimumab, Certolizumab, Infliximab have been widely used (108). For the same time, TNFai also induce RA when treating psoriasis, eczema, lupus and so on (1). The pathogenesis remains unclear. Currently, JAK inhibitors as molecular targeted compounds for rheumatoid arthritis (RA) have been marketed. Compared to biologics, JAK inhibitors have been shown to be more effective than adalimumab and abatacept in combination with MTX (109). Tofacitinib, baricitinib, upadacitinib, and filgotinib are four JAK inhibitors that have undergone Phase 3 studies. In addition, subjective measures such as pain score and patients’ global assessment witness an improvement in JAK inhibitors (110, 111). This suggests that pain and fatigue experienced by rheumatoid arthritis patients may have non-inflammation pain related to JAK-STAT pathway. In conclusion, JAK inhibitors are promised approach to replace biologics when paradoxical reaction occur.

In addition to inflammatory diseases discussed above, many other paradoxical eruptions such as sarcoidosis-like and granulomatous reactions, venous and arterial thromboembolic events have unclear mechanisms (1). Whether JAK inhibitors can be used to reduce inflammation remains unknown. Future researches should be carried out to find more clinical value of JAK inhibitors.

### 3.5 Treatment recommendation and safety issue

As a treatment recommendation, common adverse effects of JAK inhibitors should be considered cautiously. Based on the literature review, common complications for abrocitinib, upadacitinib and deucravacitinib, including infections, gastrointestinal disorders, neurological disorders, dermatologic side effects and laboratory abnormalities have been reported (97, 112–115). The most frequently reported (>5% of patients) infections include upper respiratory tract infections (URIs) and nasopharyngitis for abrocitinib, upadacitinib and deucravacitinib.

The most common gastrointestinal side effect was nausea, and another gastrointestinal side effect was diarrhea. Headaches were the most common neurologic side effect, mild and short in duration (median < 1 day) The most common dermatologic adverse events were acne and atopic dermatitis. Application site burning or pruritus were reported in < 1%. For laboratory abnormalities, complete blood count (especially neutrophil, hemoglobin, or lymphocyte counts), creatine phosphokinase and lipids were often abnormal. Similarly, JAK inhibitors prescribed to treat paradoxical reactions can also have these anticipated adverse effects, which should be treated in caution. Moreover, it is always recommended that dermatologists should consider patients “baseline risk factors” for developing serious complications when prescribing oral JAK inhibitors. The baseline risk of a particular event may be viewed as an aggregate measure of case-mix factors such as age or disease severity, such as a history of VTE, hypertension, or coronary artery disease (CAD), age > 65 years, smoking, and hormone replacement therapy/oral contraceptive use (96).

Based on literature review, risk factors of JAK inhibitors for developing complications should be assessed, including age > 65, obesity, tobacco use, cardiovascular disease, coagulation disorder, or history of thromboembolism or malignancy, indicating that careful and regular screen is necessary.

For initial screen, baseline lab test, including (1)complete blood count with a differential (2), liver and kidney function tests (3), tuberculosis test (4), Hepatitis B and C panel (5), baseline lipid panel (6), pregnancy tests should be done to exclude patients who have (1) active cancer (or history of several cancers) (2) active or recurrent shingles despite vaccination (3) severe recurrent infections and/or frequent hospitalizations for serious infections (4) previous DVT and/or high risk for DVT without receiving anticoagulation (5) pregnancy, breast-feeding, and/or patients considering pregnancy (6) receiving other immunosuppressive therapies, such as transplant patients (7) severe organ failure such as decompensated cirrhosis and end-stage renal disease requiring dialysis due to limited data in these populations.

In addition, complete blood count with a differential, liver and kidney function tests should be done one and three months after prescribing JAK inhibitors, as well as every 3-6 months later. Dose should be considered to reduce or even cease when hemoglobin, neutrophils or lymphocyte decreased considerably or liver and kidney function impaired severely. For patients inappropriate to use JAK inhibitors, glucocorticoids and cyclosporin can be used. Also, among biological agents to treat AD, dupilumab is less likely to cause paradoxical reaction. In clinical trials and long-term safety studies, only a few adult patients with moderate to severe AD have reported adverse reactions to psoriasis during treatment, and no pediatric patients have reported (33). Dupilumab selectively inhibits Th2 cytokines without affecting Th1/17/22 cytokine levels (34). Replaced with dupilumab is another potential therapy for paradoxical reaction. According to your suggestions, we have added the alternative approach for patients inappropriate to use JAK inhibitors.

In conclusion, compared with biologics, JAK inhibitors have weak specificity and can target a variety of cytokines. They are less frequent to over inhibit a certain cytokine and lead to immune dysregulation (90), which is an effective therapy for both AD and PsO, and has the potential for the treatment of paradoxical dermatoses.

## 4 Conclusion

Biologics play an active and effective role in the treatment of inflammatory dermatoses. However, paradoxical dermatoses have also emerged. A common genetic background and a common inflammatory pathway are possible mechanisms. In the face of paradoxical reactions, the choice of therapy needs to be directed toward therapies that are effective for both diseases, such as Janus kinase (JAK) inhibitors. Tofacitinib, baricitinib, ruxolitinib, abrocitinib, upadacitinib, solcitinib, itamatinib, and deucravacitinib are potential therapies for the treatment of paradoxical skin reactions. It is believed that one day in the future, JAK inhibitors will no longer be a potential treatment for paradoxical dermatoses - they are here, and the future is bright. Common side effects, baseline risk factors and safety use of JAK inhibitors were discussed.

## Author contributions

YZ: Writing – original draft. GJ: Conceptualization, Project administration, Supervision, Writing – review & editing.
